# Emerging Strategies for Cargo Loading and Engineering of Extracellular Vesicles for Breast Cancer Treatment

**DOI:** 10.3390/nano15181418

**Published:** 2025-09-15

**Authors:** Karan Mediratta, Marena D. Diab, Peter Han, Hailey Hu, Lisheng Wang

**Affiliations:** 1 The Centre for Infection, Immunity, and Inflammation (CI3), University of Ottawa, Ottawa, ON K1H 8M5, Canada; 2 Ottawa Institute of Systems Biology, University of Ottawa, Ottawa, ON K1H 8M5, Canada; 3 Department of Biochemistry, Microbiology and Immunology, Faculty of Medicine, University of Ottawa, Ottawa, ON K1H 8M5, Canada

**Keywords:** extracellular vesicles (EVs), exosomes, therapeutic cargo loading, EV engineering/functionalization, drug delivery systems, targeted therapies, breast cancer stem cells (CSCs), immunotherapy, lipid nanoparticles (LNPs)

## Abstract

Breast cancer has now surpassed lung cancer as the leading cause of cancer-related deaths among women worldwide. Given the urgent need for more effective treatment, extracellular vesicles (EVs) have gained attention as versatile and promising drug delivery systems. Derived from a variety of cell types, EVs can be loaded with therapeutic cargo or engineered to present specific surface ligands and receptors. These EV modifications enable them to overcome many limitations associated with conventional therapies. In this review, we highlight current methodologies for loading small molecule drugs, RNA-based therapeutics, and proteins into EVs through both pre-isolation (endogenous) and post-isolation (exogenous) methods. We further discuss recent advances in EV surface engineering strategies aimed at improving tumor-specific targeting and immunotherapeutic efficacy in breast cancer.

## 1. Introduction

Breast cancers represent the largest global contributor of cancer-related deaths among women, accounting for approximately 666,000 deaths recorded in 2022 [1]. Triple-negative breast cancer (TNBC) is particularly formidable, given that its immunological heterogeneity limits the successes of immunotherapies that have shown promise in several other cancer types [2,3,4,5]. Standard chemotherapeutics could further exacerbate the problem, often enriching disease-promoting cancer stem cells (CSCs) that are capable of self-renewal to drive tumor recurrence [6,7]. Despite an expanded therapeutic toolkit with advances in small molecule drugs, mRNA/proteins, small interfering RNA (siRNA), and microRNA (miRNA), achieving efficient, CSC-targeted delivery within the tumor microenvironment remains a significant translational hurdle.

In this context, extracellular vesicles (EVs) have emerged as promising drug delivery platforms that are reshaping strategies for cancer therapy. EVs are nanosized vesicles with a lipid-bilayer secreted from cells that lack functional nuclei, and unlike cells, cannot replicate on their own [8]. According to the minimal information for studies of extracellular vesicles (MISEV), EV classification by origin is discouraged, wherein EVs are described as exosomes (EVs that originate from internal compartments), ectosomes/microvesicles (EVs that originate from shedding of the cell membrane), or apoptotic bodies (EVs that originate from programmed cell death) [8,9,10]. EVs may also be categorized by size as small EVs (<200 nm in diameter) and large EVs (>200 nm in diameter) [8,11]. Functionally, EVs are central mediators of intercellular communication, transporting various biomolecules such as nucleic acids, proteins/enzymes, lipids, and metabolites to recipient cells [12,13]. Their intrinsic biocompatibility [14,15], ability to cross biological barriers [16,17,18], amenability to cargo loading and surface engineering [19,20], and scalability potential as off-the-shelf treatments [21,22,23,24] make them attractive candidates for targeted therapeutic delivery.

In parallel, lipid nanoparticles (LNPs) are nanosized synthetic delivery platforms for nucleic acids and small molecule drugs. LNPs exhibit robust formulation flexibility, incorporating ionizable or cationic lipids, sterols, phospholipids, and polyethylene glycol (PEG) into self-assembled, cargo-encapsulating vesicles [25]. LNPs gained widespread attention and clinical validation during the COVID-19 mRNA vaccine deployment, but has also shown to successfully encapsulate larger nucleic acids, proteins, and small molecule drugs [25,26,27]. Importantly, hybrid delivery systems combining EVs and LNPs are being developed to leverage the scalability and encapsulation efficiency of LNPs with the intrinsic biocompatibility and low immunogenicity of EVs in breast cancer treatment.

In this review, we provide an in-depth overview of current strategies for loading diverse therapeutic cargo into EVs, both pre-isolation (endogenous) and post-isolation (exogenous). We further discuss the advantages and limitations of each approach and highlight the potential clinical significance of EV-mediated cargo delivery in targeting the tumor microenvironment.

## 2. Post-Isolation (Exogenous) Cargo Loading in Extracellular Vesicles

**Figure 1:**
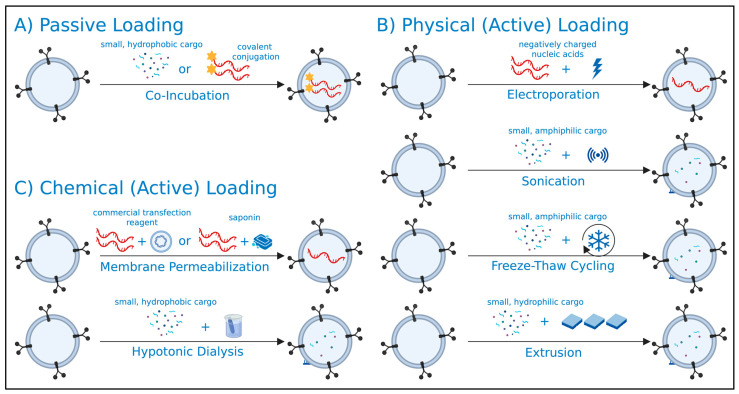
**General overview of post-isolation (exogenous) extracellular vesicle (EV) loading strategies.** EVs isolated from producer cells may undergo (**A**) passive, (**B**) active physical, or (**C**) active chemical loading strategies for the encapsulation of therapeutic cargo into the EV lumen. Created in BioRender. Mediratta, K. (2025) https://BioRender.com/6jpo955 (accessed on 5 September 2025).

Exogenous loading refers to the direct incorporation of therapeutic cargo into the EV lumen after their isolation from producer cells (Figure 1). This approach bypasses the need to alter the biology of EV producer cells by genetic or pharmacological means for induced expression of therapeutic cargo.

Post-isolation cargo loading can be achieved through passive or active methods, each having their own advantages and considerations (Table 1). Active cargo loading can be further categorized into physical and chemical methods, as outlined below. A critical consideration in post-isolation cargo loading is the requirement for additional purification steps to remove unencapsulated cargo. These steps often depend on the physical and chemical properties of the cargo, introducing additional complexities in generating uniform, cargo-loaded EV preparations [28].

### 2.1. Strategies for Passive Cargo Loading into EVs

#### 2.1.1. Co-Incubation of Cargo with EVs

Passive cargo loading relies on the diffusion of lipophilic therapeutics across the EV lipid bilayer, typically achieved through simple co-incubation methods. For example, Sun et al. passively loaded curcumin (an agent known to target upregulated signaling pathways in TNBC) into EVs derived from EL-4 T cell lymphoma cells [29]. Similarly, the chemotherapeutic agents doxorubicin and paclitaxel were incorporated into EVs derived from U-87 MG glioblastoma cells by a 2-h incubation at 37 °C [30], and into bovine milk-derived EVs through overnight incubation at room temperature [31]. The latter paclitaxel-encapsulated EVs exhibited significant cytotoxicity against human MDA-MB-231 TNBC cells. Similarly, Peng et al. encapsulated doxorubicin into EVs derived from mouse 4T1 breast cancer cells through overnight incubation at room temperature [32]. Beyond small molecules, co-incubation enabled the loading of miR-145 into EVs derived from human liver stem cells, yielding a 100-fold increase in miR-145 levels in renal CSCs relative to unloaded EVs [33]. Collectively, these studies suggest that passive post-isolation loading can preserve EV integrity while offering a standardized approach for encapsulating lipophilic chemotherapeutics.

#### 2.1.2. Covalent Conjugation-Mediated Improvements for Co-Incubation

While passive cargo loading primarily favors lipophilic therapeutics, several studies have reported that covalent conjugation of small hydrophilic siRNA and miRNAs with lipophilic moieties can enable their passive encapsulation into EVs. Lipid anchors like cholesterol [35,36,37,38,39] and vitamins [40] conjugated to the oligonucleotide backbone have been shown to enhance membrane permeability. For instance, Gong et al. successfully loaded cholesterol-modified miR159 into EVs derived from THP-1 monocytes to target the Wnt signaling pathway, which is commonly upregulated in MDA-MB-231 human TNBC cells [37]. Similarly, Cholesterol-conjugated siPLK1 encapsulated in bovine milk-derived EVs significantly suppressed HCT116 colorectal tumor growth following systemic tail vein administration [41]. In a TNBC model, cholesterol-conjugated siSurvivin encapsulated in HEK293-derived EVs effectively suppressed MDA-MB-468 tumor progression [42]. Across these studies, the ratios of cholesterol-to-siRNA and cholesterol-conjugated siRNA-to-EV are critical parameters of loading efficiency and therapeutic efficacy.

### 2.2. Physical Strategies for Active Cargo Loading into EVs

#### 2.2.1. Electroporation

Active cargo loading is often employed for larger or hydrophilic therapeutics that cannot readily permeate the lipid bilayer of EVs. Among the physical strategies, electroporation is the most commonly used, utilizing short high voltage electrical impulses to transiently disrupt EV membranes and facilitate encapsulation of charged therapeutics like mRNA and siRNA [43]. For instance, EVs derived from MCF-7 breast cancer cells were successfully loaded with siCD44 for targeted gene silencing within the same cell line [44], thereby lowering a key marker of stemness among aggressive breast cancers [71]. In another study, EVs from bone marrow-derived mesenchymal stem cells (MSCs) were electroporated with LNA-anti-miR-142-3p, selectively targeting MCF-7 breast CSCs [45]. MDA-MB-231 TNBC tumors were also targeted in vivo by EVs functionalized with the targeting aptamer T-AS1411 and loaded with let-7 miRNA by electroporation [46]. Despite its widespread use, electroporation is associated with nucleic acid aggregation, leading to overestimated loading efficiencies [47,48]. Notably, the addition of EDTA to the electroporation buffer has been reported to mitigate siRNA aggregation [47]. Beyond small RNAs, Lamichhane et al. demonstrated that DNA up to 750 bp could be efficiently loaded into HEK293-derived EVs and subsequently delivered to recipient HEK293 cells, highlighting the potential of electroporation for larger genetic payloads [48]. Moreover, electroporation has been used to load doxorubicin into EVs derived from immature mouse dendritic cells for targeted delivery to MDA-MB-231 TNBC tumors in vivo [49].

#### 2.2.2. Sonication

Sonication is another physical method for post-isolation cargo loading, wherein high frequency sound waves induce transient pores in the EV membrane, facilitating cargo entry. Sonication has been widely employed to load chemotherapeutic agents such as paclitaxel [50,51,52] and doxorubicin [51,53,54], resulting in enhanced drug accumulation and improved targeting of murine breast tumor models compared to free drug administration. The successful encapsulation of lipophilic paclitaxel and amphiphilic doxorubicin highlights the versatility of sonication-mediated cargo loading [51]. Farouk et al. also reported the loading of vincristine sulfate into MSC-derived EVs by sonication, enabling targeted delivery to T-47D breast CSCs [55]. Beyond small molecules, sonication has also been used to load HER2-targeting siRNA, with significantly reduced siRNA aggregation compared to electroporation [56]. Similarly to high frequency sound waves, microwave radiation may also be employed for the loading of small molecule drugs [57]. However, sonication may irreversibly alter the EV membrane integrity or the expression of surface proteins. For example, sonication-mediated doxorubicin loading significantly reduced expression of tetraspanin EV markers CD9 and CD63 [58]. These potential alterations may impair EV uptake by recipient cells and downstream functionality, warranting careful consideration when selecting sonication for cargo loading.

#### 2.2.3. Freeze–Thaw Cycling

Cargo loading can be achieved by freeze–thaw cycling, which involves repeated freezing of EVs at −80 °C followed by thawing at room temperature. This process relies on ice crystal formation to transiently disrupt the EV membrane for cargo entry [59]. Compared to electroporation and sonication, freeze–thaw cycling has generally reported to yield lower loading efficiencies [43,53,60]. In one study, doxorubicin was found primarily adhered to the EV surface rather than encapsulated after three freeze–thaw cycles. Subsequent purification steps to remove unencapsulated drug revealed substantially reduced encapsulation efficiency [61]. In contrast, Wu et al. reported that doxorubicin loaded into platelet-derived EVs by freeze–thaw cycling yielded improved loading efficiency and sensitized MDA-MB-231 human TNBC cells to treatment [53]. Using a modified freeze–thaw protocol, Tran et al. effectively loaded aspirin in MDA-MB-231 cell-derived EVs and demonstrated its efficacy in targeting CSCs and tumorsphere formation relative to free aspirin [62]. Freeze–thaw cycling has also been used to load miR-16-5p mimics into EVs derived from adipose MSCs using a liposome-assisted approach, which led to significantly reduced mouse 4T1 breast tumor masses in vivo [63]. Overall, conflicting reports on the efficacy of freeze–thaw cycling for EV cargo loading relative to other physical methods like electroporation and sonication warrants further investigation.

#### 2.2.4. Extrusion

Extrusion is an infrequently used post-isolation method for cargo loading, in which the EVs and free cargo are repeatedly forced through membrane filters. This process relies on the shear force generated to transiently disrupt the EV membrane, facilitating cargo encapsulation. In one report, EVs derived from MDA-MB-231 TNBC cells were loaded with hydrophilic porphyrins by extrusion, resulting in superior cytotoxicity effects relative to co-incubation, electroporation and hypotonic dialysis (a chemical strategy) [34]. Similarly, NK cell-derived EVs extruded with docetaxel exhibited improved cytotoxicity against A549 human lung cancer cells compared to NK cell-derived EVs alone, suggesting an effective delivery platform [64]. In addition to loading doxorubicin into the lumen of MCF-7 cell-derived EVs, Kim et al. reported the use of extrusion for functionalizing the EV surface with vascular endothelial growth factor receptor-1 (VEGFR1)-targeting peptide, implying dual utility of this method for both cargo loading and surface engineering [65]. Relative to other physical strategies for cargo loading, extrusion is also more amenable to large-volume workflows for potential commercialization [72]. In contrast, extrusion may alter EV size distribution, surface charge, and biological activity [66], highlighting the need for further optimization and characterization.

### 2.3. Chemical Strategies for Active Cargo Loading into EVs

#### 2.3.1. Membrane Permeabilization

Chemical methods of post-isolation cargo loading are often employed when hydrophilic cargo cannot efficiently cross the lipid bilayer of EVs. In such cases, membrane permeabilization can be facilitated using transfection reagents or saponin. Transfection reagents such as lipofectamine contain positively charged lipids that form complexes with negatively charged nucleic acids to promote their incorporation into EVs. For example, Shtam et al. effectively loaded siRNA labeled with Alexa fluor 488 into EVs using lipofectamine, achieving functional gene knockdown in recipient cancer cells [67]. However, a significant limitation of this approach is the potential of co-purification of transfection reagent complexes with EVs, given their overlapping physical characteristics [68]. Supporting this concern, one study detected Exo-Fect (a commercial transfection reagent for EVs) alongside miRNA-loaded EVs after purification, highlighting the challenge of eliminating transfection reagent complexes as residual contaminants downstream [69].

In addition to commercial transfection reagents, plant-derived steroids known as saponins have been repurposed as mild detergents to facilitate the loading of hydrophilic cargo into the EV lumen. By interacting with EV membrane cholesterol, saponins can transiently disrupt the EV membrane for cargo encapsulation. Fuhrmann et al. demonstrated that 0.01% (w/v) saponin significantly enhanced encapsulation of porphine benzoic acid into MDA-MB-231-derived EVs relative to electroporation- and extrusion-mediated EV loading [34]. In another study, doxorubicin was encapsulated into EVs derived from U937 monocytes using 0.2% saponin. These doxorubicin-loaded EVs exhibited nearly identical cytotoxicity curves to free doxorubicin in recipient cell lines [61]. Conversely, Abreu et al. reported that saponin was ineffective for loading miR-155 into human urine-derived EVs [69]. Similarly to transfection reagents, saponin poses the risk of residual contamination, which may interfere with downstream applications [70].

#### 2.3.2. Hypotonic Dialysis

A less commonly employed chemical method for EV loading is hypotonic dialysis, which involves co-incubating EVs with cargo in a hypotonic buffer to induce osmotic swelling and transient pore formation in the EV membrane for cargo entry [70]. Loaded EVs are then returned into an isotonic solution to restore EV membrane integrity. In addition to co-incubation, electroporation, and extrusion, Fuhrmann et al. also investigated hypotonic dialysis as a loading method; however, they found it resulted in very poor drug release for targeting MDA-MB-231 TNBC cells [34]. Beyond this, use of hypotonic dialysis for drug loading in breast cancer application remains limited and largely underexplored.

## 3. Pre-Isolation (Endogenous) Cargo Loading of Extracellular Vesicles

**Figure 2:**
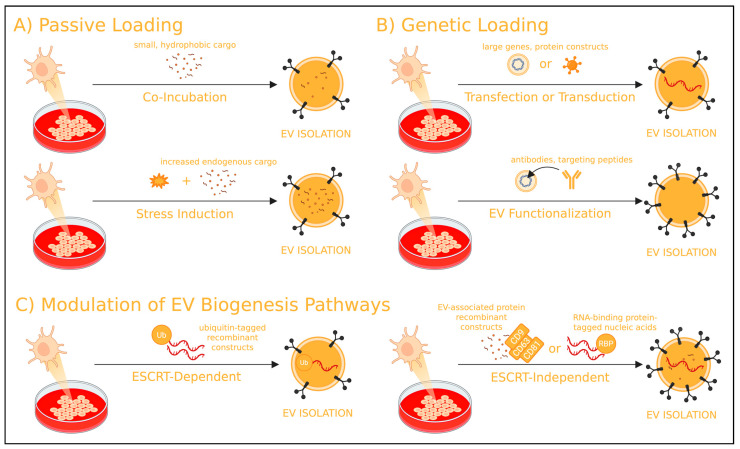
General overview of pre-isolation (endogenous) EV loading strategies. EV producer cells may undergo (**A**) passive, (**B**) genetic, or (**C**) modulation of EV biogenesis loading strategies for the encapsulation of therapeutic cargo into subsequently isolated EVs. Created in BioRender. Mediratta, K. (2025) https://BioRender.com/h4372c5 (accessed on 5 September 2025).

Endogenous loading involved introducing therapeutic cargo directly into EV producer cells prior to vesicle secretion (Figure 2). This approach eliminates the risk of disrupting the EV membrane and the need for downstream purification to remove unencapsulated cargo.

Endogenous loading can be achieved through passive co-incubation, genetic engineering of EV producer cells, or modulation of EV biogenesis pathways—each offering distinct advantages and considerations (Table 2). EV biogenesis can occur via either the endosomal sorting complex required for transport (ESCRT)-dependent pathway or ESCRT-independent pathway, both of which are described below.

### 3.1. Passive Strategies for Cargo Loading in EV Producer Cells

#### 3.1.1. Co-Incubation of Cargo with Producer Cells

Similarly to passive co-incubation methods applied after EV isolation, a simple pre-isolation approach involves co-incubating lipophilic drugs with EV producer cells. By leveraging the cell machinery for endogenous EV loading, this method has been employed in several studies with chemotherapeutic agents such as paclitaxel [73,74] and doxorubicin [51,75]. For instance, Farhat et al. showed that breast cancer cell lines 4T1 and SKBR3 incubated with doxorubicin under normal culture conditions produced doxorubicin-loaded EVs without altering their biophysical properties compared to unloaded EVs [75]. Drug loading efficiency may also be enhanced through extrusion of producer cells, as shown by Kalimuthu et al., who extruded paclitaxel with human MSCs, yielding paclitaxel-loaded EVs with potent in vivo efficacy against MDA-MB-231 TNBC tumors [76]. In addition to cargo loading, Zhu et al. demonstrated that EV producer cells can be co-incubated with 1% DSPE-PEG-Folate or DSPE-PEG-Biotin to functionalize EV membranes, improving targeted delivery to MDA-MB-231 TNBC cells [77]. This technique was also used to load the tumor-targeting cRGD peptide onto MSC-derived EVs [78]. DSPE-PEG is a widely accepted amphiphilic drug delivery platform with the ability to integrate efficiently into lipid bilayer [129], making it a versatile tool for EV surface engineering.

#### 3.1.2. Inducing Stress on Producer Cells

Several reports have demonstrated that specific treatments and culture conditions applied to EV producer cells can enhance both EV yield and therapeutic cargo loading efficiency. For example, culturing EV producer cells with the cholesterol metabolite 27-hydroxycholesterol significantly increased EV secretion in polymorphonuclear neutrophils, RAW264.7 monocytes, and mouse 4T1 breast cancer cells [79]. In another study, MDA-MB-231 TNBC cells treated with SKF96365, an inhibitor of intracellular calcium signaling, exhibited increased EV production with elevated miR-145 levels [80]. Interestingly, this contrasts with a report from Sako et al., in which activating calcium signaling increased EV secretion from mouse bone marrow-derived dendritic cells [81]. In addition to treatment with small molecules, physical stimuli may also influence EV biogenesis. Exposure to ultraviolet (UV) light has been reported to increase EV production or improved loading of small molecule drugs [82,83,84]. Similarly, heat stress has been found to increase doxorubicin loading into MCF-7-derived EVs, which subsequently inhibited MCF-7 breast tumor growth in vivo [85]. Exposing EV producer cells to paclitaxel has also been reported to enhance EV secretion in human hepatocarcinoma HepG2 cells, and increase the encapsulation of heat shock proteins [73]. Moreover, EVs derived from paclitaxel-treated human MSCs exhibited enhanced targeting of MDA-MB-231 TNBC cells, attributed to the enrichment of several endogenous miRNAs [86]. These findings align with broader evidence indicating that cancer cells release more EVs when exposed to chemotherapeutic agents [87,88].

### 3.2. Genetic Strategies for Cargo Loading in EV Producer Cells

#### 3.2.1. Transfection or Transduction of Producer Cells

While passive cargo loading has proven effective for incorporating lipophilic chemotherapeutics, genetic cargo such as RNA is typically over-expressed in EV producer cells to enable subsequent endogenous encapsulation. Several reports have demonstrated the successful transduction of MSCs to over-express therapeutic miRNAs such as miR-379 [89], miR-34a [90], and miR-124a [91], all capable of suppressing breast cancer cell lines in vitro and in vivo. In addition, human MSCs may naturally express anti-tumor miRNAs. For example, Pakravan et al. identified the endogenous expression of miR-100, which suppressed the HIF-1ɑ/mTOR signaling pathway in MDA-MB-231 TNBC cells [92]. EVs derived from HEK293FT transfected with HChrR6 mRNA encoding a bacterial enzyme selectively targeted HER2-positive BT474 breast cancer cells [93]. In a separate study, HEK293 cells transfected with mIR-HER2-E1 produced EVs that effectively targeted HER2-postive SK-OV-3 breast cancer cells [94]. Interestingly, PTEN mRNA, commonly used to block the PI3k/Akt pathway in CSCs, was effectively encapsulated in EVs from cellular nanoporation, a technique developed by Yang et al. [95]. They transfected various producer cells with plasmid DNAs and stimulated the cells with a focal and transient electrical stimulus to promote the release of EVs carrying transcribed mRNAs and targeting peptides. These findings underscored the potential of EVs as carriers of genetic therapeutics. However, the choice of producer cell line, transfection efficiency, and maintenance of cell viability/health remain major considerations for successful application.

#### 3.2.2. EV Surface Functionalization

Genetic modification of EV producer cells can also be employed to functionalize EVs with targeting peptides, enhancing their specificity toward breast cancer cells. In a study by Ohno et al., HEK293 cells were transfected with the EGFR-specific GE11 synthetic peptide using the pDisplay vector, resulting in EVs with higher binding affinity to recipient HCC70 TNBC cells that express higher levels of EGFR in vitro and in vivo [96]. In the same study that loaded HChrR6 mRNA, Wang et al. further transfected HEK293FT cells with a synthetic construct (pEVC1C2HER), enabling the isolated EVs to target HER2-positive breast cancer cells [93]. Similarly, the HER2-specific Designed Ankyrin Repeat Protein (DARPin) ligand was stably expressed on HEK293 cells via lentiviral transduction, and its derived EVs effectively targeted HER2-positive SKBR3 breast cancer cells [97]. Another approach involved the expression of anti-HER2 Trastuzumab light chains 1 and 2 fused to CD81 and tagged with a GFP reporter in HEK293T cells, yielding EVs capable of targeting HER2-expressing breast cancer cells [98].

EV functionalization strategies can also involve the expression of high affinity ligands designed to competitively inhibit tumor-promoting receptors. For instance, Koojimans et al. transfected producer cells with the Ega1 nanobody (a competitive ligand for EGFR) fused C-terminal to a GPI signal peptide, yielding EVs capable of blocking EGFR signaling in recipient HeLa and A431 tumor cells [99]. In another approach, human MSCs were engineered to express the tumor necrosis factor-related apoptosis-inducing ligand (TRAIL), resulting in the production of EVs capable of inducing cytotoxicity against MDA-MB-231 TNBC cells [100]. HEK293F cells were also engineered to co-express EGFR and CD47, generating EVs with enhanced ability to target TNBC patient-derived xenograft tumors [101]. Additionally, functionalization with an iRGD-Lamp2b fusion construct significantly improved EV-mediated delivery of doxorubicin to ɑv integrin-expressing breast cancer cells [49].

### 3.3. Modulating EV Biogenesis Pathways for Cargo Loading in EV Producer Cells

#### 3.3.1. ESCRT-Dependent Pathway Modulation

**Figure 3:**
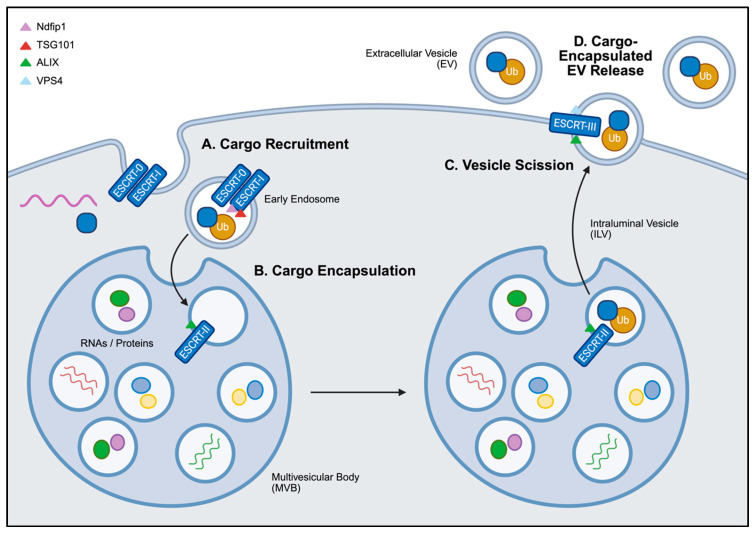
**General overview of ESCRT-dependent EV cargo loading.** (**A**) Ubiquinated proteins are recognized by ESCRT-0 and ESCRT-I and recruited to the membrane of early endosomes, facilitated by Ndfip1 and TSG101. (**B**) These endosomes subsequently mature into intraluminal vesicles (ILVs) within multivesicular bodies (MVBs). (**C**) With the help of ALIX and the ATPase VPS4, ESCRT-III drives the final assembly and membrane scission of ILVs from the MVBs. (**D**) The fusion of MVBs with the cell membrane drives the release of cargo-encapsulated ILVs, referred to as EVs in the extracellular space. Created in BioRender. Mediratta, K. (2025) https://BioRender.com/bibp7z3 (accessed on 5 September 2025).

The endosomal sorting complexes required for transport (ESCRT) machinery play a central role in EV biogenesis, particularly in the sorting and loading of ubiquinated proteins (Figure 3). The four pathway subcomplexes, denoted ESCRT-0, ESCRT-I, ESCRT-II, and ESCRT-III work sequentially to facilitate the internal budding of intraluminal vesicles within multivesicular bodies. ESCRT-0 through ESCRT-II mediate recognition and recruitment of cargo from the cytoplasm to the endosomal membrane, while ESCRT-III drives vesicle cleavage and final assembly [130]. Several accessory components further regulate the ESCRT-dependent pathway, including VPS4 (Vacuolar Protein Sorting-associated Protein 4), an ATPase that powers the recycling of ESCRT-III [131], ALIX (Apoptosis-Linked Gene 2-Interacting Protein X) that recruits ESCRT-III proteins to the cell membrane [132], Syntenin that works with ALIX to recruit specific cargo [102], Ndfip1 (Nedd4 Family-Interacting Protein 1) that recruits ubiquitin ligases to promote cargo recognition [133], and TSG101 (Tumor Susceptibility Gene 101) that engages ubiquinated cargo for ESCRT-I [134]. It is important to note that EV biogenesis still occurs in the absence of ESCRT machinery [135].

Although direct applications of ESCRT modulation for EV loading are limited, adding tags to recombinant constructs provide an avenue to exploit ESCRT machinery. For example, adding a ubiquitin tag to the C-terminus of a recombinant protein in HEK293 cells resulted in a ten-fold increase in EV cargo loading [103]. However, concerns remain regarding the transient nature of ubiquitination, as cargo may undergo deubiquitylation prior to being loaded into EVs [104]. Alternatively, the addition of two WW-domain tags to Cre recombinase enabled its efficient loading via Ndfip1-mediated recognition and ubiquitination [105]. These strategies highlight the potential to harness ESCRT-dependent pathways for the loading of breast cancer-targeting proteins into EVs.

In addition to EV loading, the ESCRT-dependent pathway has also been explored as targets for inhibiting tumor-associated EV secretion. For example, overexpression of the small GTPase RAB22A in MDA-MB-231 TNBC cells was shown to enhance EV biogenesis under hypoxic conditions, confirming its role in recruiting proteins to the membrane of early endosomes [106,136]. Moreover, Datta et al. demonstrated that blocking ESCRT-0 proteins in PC-3 prostate cancer cells using Manumycin A suppressed EV secretion [107]. Similarly, significantly reduced EV production was observed from the silencing of TSG101 in HeLa cells [108], and ALIX and associated components in MCF-7 cells [102]. These findings highlight the conserved role of ESCRT-associated proteins in EV biogenesis across multiple cell types, including breast cancers.

#### 3.3.2. ESCRT-Independent Pathway Modulation

**Figure 4:**
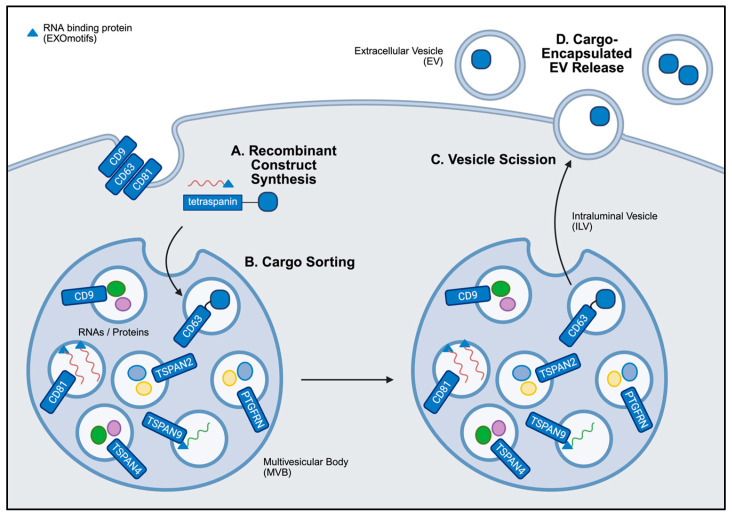
**General overview of ESCRT-independent EV cargo loading.** (**A**) The synthesis of recombinant proteins by endogenous cell machinery enables cargo to be generated by EV producer cells. (**B**) These recombinant constructs encoding tetraspanins, RNA-binding proteins, or lipid raft-associated motifs are sorted into intraluminal vesicles (ILVs) within multivesicular bodies (MVBs) during EV biogenesis. Cargo may be attached to the tetraspanins in the lumen or external to the ILV, depending on the construct design. (**C**) These variables may influence the rate of vesicle scission. (**D**) The fusion of MVBs with the cell membrane drives the release of cargo-encapsulated ILVs, referred to as EVs in the extracellular space. Created in BioRender. Mediratta, K. (2025) https://BioRender.com/31nzfne (accessed on 5 September 2025).

Pathways independent of ESCRT also play a significant role in EV biogenesis and cargo loading (Figure 4). Key components of these pathways include tetraspanins, sphingomyelins, ceramides, and lipid raft domains. Tetraspanins such as CD9, CD63, and CD81 function to shuttle proteins and lipids into intraluminal vesicles, the precursor of EVs. Given that increased expression of tetraspanins have been associated with increased EV secretion in breast cancer cells [137,138,139], researchers may find utility in recombinant proteins using tetraspanins as scaffolds for engineered cargo loading.

Initial demonstrations of this approach involve fusing tetraspanins with GFP to track EV biodistribution [109,110], and with ovalbumin to promote antigen-specific immune priming [111]. While no direct applications in breast cancer have been reported, several related studies offer relevant insights. For instance, Bellavia et al. engineered a fusion construct combining an IL-3 fragment with the EV-associated Lamp2B protein to selectively target IL-3R-expressing chronic myeloid leukemia cells [112]. Alternatively, fusing the tumor suppressor p53 to CD63 in HEK293T-derived EVs enabled delivery to H1299 human non-small cell lung carcinoma cells, resulting in significantly increased intracellular p53 protein levels and enhanced apoptosis [113]. Innovative approaches have also employed inducible systems to control EV cargo loading and release. In a study by Choi et al., the light-inducible CIBN/CRY2 protein interaction system was used to load the NF-κB inhibitor super-repressor IκB in EVs. CIBN was fused to CD9 on the EV membrane, while CRY2 was fused to the cargo protein in a separate recombinant construct [114]. Similarly, Cas9 was successfully delivered from transfected HEK293FT-derived EVs using light-sensitive stoplight reporter cells [115]. Ivanova et al. introduced a self-cleaving DnaB helicase Intein to release Cre recombinase from a tetraspanin fusion construct within the EV lumen [116]. Similarly, Zuppone et al. used a tumor-specific Cathepsin b cleavage site to trigger the release of RFP protein from the recombinant construct once delivered to target cells [117].

Beyond the delivery of therapeutic proteins, the efficient encapsulation of oligonucleotides and RNA-based therapeutics often requires different fusion strategies. In a study by Es-Haghi et al., fusing the tetraspanin CD9 with the RNA-binding protein AGO2 significantly increased the levels of endogenously expressed miRNAs within the EVs [118]. In a separate study, the murine OX40L mRNA was successfully delivered to B16F10 melanoma tumors in vivo using a recombinant construct combining an RNA-binding protein with CD63 [119]. Among RNA-binding proteins, the heterogenous nuclear ribonucleoprotein A1 (hnRNPA1) has emerged as a widely utilized scaffold in EV engineering, given its ability to recognize short RNA sequences known as EXOmotifs [120,121,122,123]. Notably, a bioinformatics analysis found strong similarities between EXOmotifs and lipid raft-associated motifs [124]. Lipid rafts are clusters of enriched cholesterol and sphingolipids found on the EV membrane [125,128], which may facilitate the binding of therapeutic RNAs. These insights have prompted the development of recombinant constructs that incorporate RNA-binding proteins and lipid raft-associated motifs to enhance RNA loading efficiency [41,126,127,128].

Exploring additional aspects of tetraspanins in recombinant proteins offer several benefits for targeting breast cancer. For example, Curley et al. demonstrated that specific truncated forms of CD63, rather than the full-length protein, sufficiently enabled cargo loading in HEK293-derived EVs [140]. Furthermore, the insertion of albumin-binding domains into the extracellular loops of several tetraspanins significantly extended the circulation time of EVs in vivo [141]. In addition to the canonical tetraspanins, recent studies have identified alternative scaffold proteins, including TSPAN2, TSPAN4, TSPAN9, and PTGFRN (Prostaglandin F2 Receptor Negative Regulator), as more effective platforms for EV cargo encapsulation [142,143,144]. These recent advances highlight the growing clinical potential of engineered EVs, wherein recombinant proteins facilitate efficient cargo loading, enhanced bioavailability, and targeted delivery for breast cancer therapy.

## 4. EV Surface Functionalization and Immunotherapy

In the context of EV cargo loading and functionalization for immunotherapy, both pre-isolation and post-isolation techniques may be employed. Given the immunosuppressive nature of breast cancer-derived EVs [80,145,146,147,148,149], we explore the therapeutic potential of EVs engineered to boost anti-tumor immunity via modification of T cells, NK cells, and tumor-associated macrophages (TAMs), as summarized in Table 3.

### 4.1. Engineered EVs and T Cells

A growing body of research highlights the potential of engineered EVs to enhance T cell-mediated anti-tumor immunity in breast cancer. In one case, the co-incubation of cGAMP (a STING agonist) with HEK293T-derived EVs at room temperature for 16 h enabled its successful encapsulation. These cGAMP-loaded EVs significantly reduced B16F10 tumor growth while promoting CD8^+^ T cell activation in vivo [150]. Similarly, co-incubation of human tumor-derived endothelial cells with anti-IL-3R led to the secretion of anti-IL-3R-expressing EVs that effectively reduced the cell migration capacity of MDA-MB-231 TNBC cells [151]. In another study, PD-1-expressing HEK293T-derived EVs were electroporated post-isolation to load the immune adjuvant imiquimod. These EVs reduced CD8^+^ T cell exhaustion and improved targeting of mouse 4T1 breast tumors [152]. Interestingly, native bacterial-derived EVs combined with anti-PD-1 [154], as well as bacterial-derived EVs engineered to express PD-1 [153], both improved tumor-infiltrating CD8^+^ T cell responses.

Furthermore, EVs engineered to express co-stimulatory molecules have demonstrated synergistic immune activation. In the study by Ji et al., CD62L and OX40L were transduced into DC2.4 mouse dendritic cells, generating EVs that co-expressed these ligands to enhance T cell effector functions in mouse 4T1 breast tumor models [155]. In a separate approach, platelet-derived growth factor receptor (PDGFR) was used as a membrane scaffold for fusing anti-CD3 and anti-EGFR in a synthetic construct to transfect Expi293F cells [156]. The resulting EVs were used to target EGFR-expressing MDA-MB-468 TNBC tumors in vivo. A follow-up study extended this strategy by generating anti-CD3 and anti-HER2-expressing EVs that enhanced T cell activation while targeting HER2-positive HCC1954 breast cancer tumors [157]. Interestingly, CAR-T cells have also been explored as EV producer cells. Fu et al. derived EVs from mesothelin-targeting CAR-T cells that also expressed the CAR construct and exhibited reduced side effects than conventional CAR-T therapy [158]. Mesothelin-targeting CAR-T cell-derived EVs were also effective at reducing the growth of BT-549 and mesothelin-expressing MDA-MB-231 TNBC tumors [159]. Collectively, these strategies highlight the potential of engineered EVs to modulate T cell activity and enhance anti-tumor immunity within the breast tumor microenvironment.

### 4.2. Engineered EVs and NK Cells

Rather than focusing on NK-mediated anti-tumor immunity, many studies investigate the use of NK cells as EV producers. In a study by Kaban et al., BCL-2-targeting shRNA was transduced into NK-92MI cells, generating EVs that induced apoptosis in MCF-7 breast cancer cells [160]. In another study, the photosensitizer Chlorin e6, known to generate pro-inflammatory ROS that promotes M1-like macrophage polarization, was loaded into NK-derived EVs via both co-incubation and electroporation [161]. Owing to its hydrophobicity, Chlorin e6 exhibited similar loading efficiency with both methods. CAR-NK cells have also been used to produce functionalized EVs. In one study, EVs isolated from CAR-NK cells were modified to express the T7 transferrin receptor binding peptide using DSPE-PEG, inducing ferroptosis in a HER2-positive brain metastatic breast cancer tumor model in vivo [162]. In another study, Huang et al. transduced NK-92MI cells to overexpress NKG2D alone or co-expressed with IL-24. The combination approach induced significantly enhanced apoptosis in both HeLa and MCF-7 breast cancer cells [163]. Interestingly, several studies have reported that NK-derived EVs themselves possess intrinsic anti-tumor activity [167,168,169,170,171], suggesting that complex engineering may not be an urgent avenue for harnessing their therapeutic potential.

### 4.3. Engineered EVs and Tumor-Associated Macrophages

Given the pro-inflammatory, anti-tumor functions of M1-like macrophages and the tumor-promoting roles of M2-like macrophages, much of the current research on EV engineering focuses on polarizing TAMS towards an M1-like phenotype. In one study, Zhang et al. fused the CRV peptide to Lamp2b to produce HEK293T-derived EVs capable of specifically targeting M2-like TAMs and polarizing them to an M1-like phenotype [164]. Alternative to genetic modification on EV producer cells, EV surface functionalization can also be achieved through chemical conjugation methods such as click chemistry. For example, Nie et al. initially polarized RAW264.7 macrophages to an M1-like phenotype using manganese (II) chloride, then functionalized the M1-derived EVs with CD47 and SIRPɑ antibodies using dibenzocyclooctyne (DBCO). These engineered EVs effectively reprogramed M2-like TAMs to an M1-like phenotype and simultaneously targeted 4T1 breast tumors in mice [165]. In a similar approach, M1-derived EVs were decorated with IL-4R-targeting peptide using a phospholipid membrane anchor and loaded post-isolation with NF-κB p50 siRNA and miR-511-3p. The resulting EVs had enhanced ability to target and reprogram TAMs in 4T1 breast tumors [166]. These approaches offer more flexible and time-efficient alternatives to genetically manipulate EV producer cells for EV surface engineering.

## 5. EV/Lipid Nanoparticle Hybrid Systems

LNPs have also emerged as powerful drug delivery systems, given their commercial and clinical trial progresses. In the context of breast cancer treatment, LNPs including liposomes, solid lipid nanoparticles (SLPs), and Lipid–Polymer Nanoparticles (LPNs) offer high stability, cargo encapsulation efficiency and sustained drug release [172,173,174]. Given their shared lipid bilayer structure, LNPs and EVs share an overlap in strategies for cargo loading and surface engineering, warranting further discussion.

### 5.1. LNP Cargo Loading

Like EVs, LNPs can be loaded passively or actively, dependent on the physiochemical properties of the cargo. The emulsion-solvent evaporation method is a commonly used passive cargo loading strategy. It involves mixing the cargo and LNP materials in a volatile organic solvent to form the oil phase and subsequently emulsifying the mixture into an aqueous phase until the solvent has evaporated [175]. Although this method is frequently used for hydrophobic cargo such as tamoxifen [176] or paclitaxel [177], a modified double emulsion method can be used for hydrophilic cargo such as doxorubicin [178] or siRNA [179,180].

### 5.2. LNP Surface Functionalization

Like EVs, the surface of LNPs can also be functionalized to enable targeted cargo delivery to breast cancer cells. In one study, Mehta et al. encapsulated siXBP1 into PLGA-based LNPs functionalized with EGFR antibodies using thiol-maleimide reactions [181]. Another study targeted integrin avβ3 receptors overexpressed in TNBC by conjugating a C-peptide onto solid LNPs, reducing 4T1 breast tumor volumes by 80% in mice [182]. The C-peptide functionalization was achieved by carbodiimide chemistry, in which activated carboxyl groups link with primary amines. Carbodiimide chemistry has been widely used in several studies for functionalizing LNPs. For example, the adenosine receptors overexpressed in TNBC was targeted by conjugating adenosine on paclitaxel-loaded LNPs using carbodiimide chemistry, resulting in increased apoptosis in MDA-MB-231 TNBC cells relative to paclitaxel alone [183]. Multi-functional LNPs have also been explored. In one study, LNPs co-loaded with gefitinib and lycorine hydrochloride were co-functionalized with iRGD and anti-HER2 trastuzumab to effectively target MCF-7 breast cancer cells [184]. Trastuzumab was attached by passive incubation following carboxyl group activation, while the iRGD peptide was conjugated by carbodiimide chemistry.

Other functionalization methods also mirror those used in EVs, such as DSPE-PEG conjugation. For instance, trastuzumab and folic acid were conjugated onto to target the folate receptors on several breast cancer cell lines, improved targeting efficacy [185]. The trastuzumab was coupled to maleimide moieties, while both folic acid and maleimide-trastuzumab conjugates were incorporated onto the LNP surface using DSPE-PEG. Beyond LNPs, various other NP platforms have demonstrated comparable targeting capabilities in TNBC [186,187,188,189,190]. Taken together, these approaches highlight the shared strategies between EV and LNP engineering, suggesting that some LNP functionalization approaches can be adapted for EVs in post-isolation modifications.

### 5.3. EV/LNP Hybrid Platforms

Given the overlap between EVs and LNPs, several studies have explored hybrid platforms that combine the advantages of both drug delivery systems to achieve synergistic therapeutic outcomes. The synthesis of hybrid platforms may occur by physical methods (co-incubation, co-extrusion, freeze–thaw cycling) or chemical methods (PEG induction) to achieve EV/LNP membrane fusion [191,192,193] or LNP cloaking using EV membranes [193,194,195]. Together, hybrid platforms strive to leverage the biocompatibility and low immunogenicity of EVs while leveraging the encapsulation efficiency and scalability of LNPs. Specifically, the expression of integrins, tetraspanins, and other surface proteins such as CD47 introduced from EV membranes onto the hybrid platforms promote cellular uptake and evasion of phagocytosis, overcoming the poor in vivo circulation of LNPs alone [18,196,197]. Simultaneously, the hybrid platforms exhibit enhanced cargo permeability and retention, overcoming the low encapsulation efficiencies of EVs alone [198,199,200]. Despite these benefits, the increased manufacturing complexity and lack of standardized protocols for generating EV/LNP hybrid platforms remain challenging.

In the context of application for breast cancer treatment, Bose et al. isolated 4T1 TNBC cell-derived EVs engineered to display the urokinase plasminogen activator (uPA) peptide and fused them with anti-miR-21-loaded LNPs via extrusion [201]. This process yielded a mixed population of EVs encapsulating anti-miR-21 and LNPs functionalized with the uPA peptide, reducing 4T1 tumor growth when administered with low-dose doxorubicin in mice. In another study, MDA-MB-231-derived EVs were conjugated with doxorubicin-loaded LNPs using the Bangham method, which involves dissolving EVs in a methanol:chloroform solution and incorporating them into a lipid phase that is subsequently evaporated [202]. These hybrid platforms exhibited enhanced cytotoxicity against both MDA-MB-231 and MCF-7 breast cancer cells, with cellular uptake levels comparable to free doxorubicin.

Choi et al. employed freeze–thaw cycling to generate a hybrid platform combining doxorubicin-loaded LNPs with EVs isolated from adipose-derived stem cells using ten cycles of rapid freezing at −80 °C followed by gradual thawing to 4 °C [203]. This hybrid platform more effectively reduced MDA-MB-231 TNBC cell viability better than both free drug and LNP-loaded doxorubicin alone. A similar approach was utilized to generate a hybrid platform of CD47-expressing EVs isolated from transduced CT26 colon carcinoma cells with indocyanine green- and Imiquimod-loaded LNPs, using only three freeze–thaw cycles to sufficiently generate the hybrid platform [197]. Additionally, hybrid EV/LNP platforms have been employed to improve immunotherapy. For instance, anti-PD-L1-loaded hybrids showed improved targeting of B16F10 tumors compared to anti-PD-L1-loaded LNPs alone [204]. In another study, Zhang et al. employed multiple strategies to achieve the loading of miR-21 into EGFR-expressing MCF-7-derived EVs fused with LNPs [205], underscoring the versatility of these hybrid drug delivery platforms for breast cancer therapy.

## 6. Limitations of EV Cargo Loading and Future Perspectives

Encapsulating various therapeutic cargo into EVs has garnered considerable traction in recent years. Despite recommendations on EV nomenclature in the MISEV guidelines, many studies continue to use inconsistent terms such as exosomes, microvesicles, or extracellular nanoparticles, often without clearly defining the isolation processes. In this review, we compiled findings from studies using varied terminologies under the umbrella term EVs. Moreover, the field of EV cargo loading and functionalization is evolving rapidly, and some aspects discussed here may be refined or replaced by future discoveries. Here, we highlight ongoing experimental challenges that continue to hinder the clinical translation of EVs as drug delivery vehicles.

An initial challenge is the incomplete understanding of EV biogenesis. As discussed in this review, many studies use a wide range of EV-producing cell lines without accounting for potential cell-specific differences in EV yield. For example, while MCF-7 breast cancer cells are frequently used to isolate EVs, Hurwitz et al. demonstrated that MCF-7 cells secrete significantly fewer vesicles per cell compared to other cancer cell lines [139], creating potential gaps between preclinical research and large-scale manufacturing. Although the kinetics of EV secretion are increasingly being investigated [206,207,208], a deeper understanding of the molecular machinery involved in EV biogenesis is still needed. Addressing the knowledge gap regarding proteins involved in ESCRT-dependent and ESCRT-independent pathways is essential to advancing pre-isolation cargo loading. Many studies employ multiple recombinant constructs using different scaffold proteins in different cell lines, making it difficult to interpret the influence of cell type on construct expression and EV cargo loading efficiency.

Secondly, enhancing the loading efficiency of EVs post-isolation is critical. Although a variety of loading techniques have been explored, there is a clear need for standardization to establish which techniques are most efficient and reproducible. Compounding this issue is the variability of methods used to separate loaded EVs from unencapsulated cargo, such as size-exclusion chromatography, ultracentrifugation, density-gradient centrifugation, ultrafiltration, or dialysis [209]. Overcoming this variability remains challenging as effective post-loading purification must be tailored to the physiochemical properties of the therapeutic cargo. Additionally, the reproducibility of post-isolation EV loading is largely dependent on the precise reporting of isolation protocol and the use of appropriate procedural controls to confirm complete removal of free cargo [210]. In contrast to our work, other reviews organize and compare the various loading strategies for the same therapeutic cargo, providing additional insight on EV loading [211]. Even with efficient loading, the rapid intracellular degradation of therapeutic cargo in recipient cells following EV uptake remains a significant barrier to sustained therapeutic efficacy [212].

Thirdly, standardized culture conditions of producer cells may improve both EV yield and clinical relevance for pre-isolation and post-isolation EV loading. Predictable and scalable EV production is essential for clinical translation and other downstream processes, as demonstrated by the work of several groups [21,213,214,215]. While this review briefly discussed the influence of cell culture conditions and cargo encapsulation strategies on EV output, batch-to-batch variability can also undermine the therapeutic efficacy of EVs. Therefore, early integration with Good Manufacturing Practice (GMP)-aligned protocols is vital to ensure reproducibility and facilitate the successful translation of EV-based drug delivery systems from bench to bedside.

## 7. Conclusions

The application of EVs as drug delivery systems has advanced considerably, leveraging their intrinsic versatility and adaptability. In this review, we outlined a range of therapeutic cargo loading strategies, each with distinct strengths and considerations largely dictated by the physiochemical properties of the therapeutic payload. EVs encapsulating small molecule drugs, RNA-based therapeutics (such as siRNA, miRNA, and mRNA), and proteins have shown remarkable improvements in preclinical models for breast cancer models. Moreover, the advances in EV surface engineering/functionalization and the development of EV/LNP hybrid drug delivery platforms offer promising avenues for achieving synergistic, multi-targeted therapeutic effects. Despite these progresses, several key challenges remain, including a deeper understanding of producer cell-specific EV biogenesis, standardizing cargo loading protocols, and ensuring batch-to-batch consistency, especially in the context of clinical translation and large-scale manufacturing. Addressing these challenges will be critical for fully realizing the therapeutic potential of EVs in the treatment of breast cancer.

## Figures and Tables

**Table 1:** Summary of advantages and considerations of post-isolation (exogenous) extracellular vesicle (EV) loading strategies.

Methods	Mechanism	Advantages	Considerations	References
**Passive**				
Co-incubation	Incubates EVs with lipophilic chemotherapeutics for passive diffusion	Non-invasive; preserves EV membrane integrity; may be improved by covalent conjugation	Limited to lipophilic drugs; poor loading efficiency	[29,30,31,32,33,34,35,36,37,38,39,40,41,42]
**Physical (Active)**				
Electroporation	Uses short electrical impulses to disrupt EV membranes for cargo entry	High loading efficiency; produces more consistent EVs	May alter EV membrane integrity; siRNA cargo aggregation	[34,43,44,45,46,47,48,49]
Sonication	Uses ultrasonic waves to disrupt EV membranes for cargo entry	High loading efficiency; applicable for hydrophilic and lipophilic cargo	May alter EV membrane integrity and surface proteins	[50,51,52,53,54,55,56,57,58]
Freeze–Thaw Cycling	Cycles of freezing and thawing to disrupt EV membranes for cargo entry	Does not require specialized equipment	Low loading efficiency; possible loss of therapeutic efficacy from cargo degradation	[43,53,59,60,61,62,63]
Extrusion	Simultaneous forcing of EVs and therapeutic cargo through membrane filters	Possibility of commercial scaling	May alter EV membrane integrity	[34,64,65,66],
**Chemical (Active)**				
Membrane Permeabilization	Uses commercial reagents like lipofectamine or plant-derived steroids like saponin to permeabilize EV membranes	Enhances loading efficiency of oligonucleotides; milder on EV membrane integrity relative to physical methods	May alter EV membrane integrity; risk of residual contamination	[34,61,67,68,69,70]
Hypotonic Dialysis	Cycles of placing EVs in hypotonic and isotonic solutions to induce osmotic swelling for cargo entry	Preserves EV membrane integrity; possibility of commercial scaling	Possibility of reduced cargo release; infrequently studied	[34,70]

Abbreviations: EVs: extracellular vesicles; siRNA: small interfering RNA.

**Table 2:** Summary of advantages and considerations of pre-isolation (endogenous) EV loading strategies.

Methods	Mechanism	Advantages	Considerations	References
**Passive**				
Co-incubation	Incubates cargo with producer cells, enabling endogenous cell machinery to release loaded EVs	Does not require potentially harsh physical or chemical methods	Heterogenous EV cargo; unpredictable loading efficiencies	[51,73,74,75,76,77,78]
Stress Induction	Inducing stress in producer cells to enhance EV secretion	Increased EV yield and cargo loading for scalability; avoids genetic manipulation of producer cells	Heterogenous EV cargo; Possible toxicity to producer cells may alter EV biogenesis	[79,80,81,82,83,84,85,86,87,88]
**Genetic**				
Transfection/Transduction	Transfecting producer cells with nucleic acid vectors to drive endogenous expression of RNA- or protein-based cargo for EV incorporation	Efficient loading for large RNA-based cargo	Possible toxicity to producer cells may alter EV biogenesis; variable transfection efficiencies	[89,90,91,92,93,94,95]
EV Functionalization	Using nucleic acid vectors to induce expression of a targeting ligand/receptor on EV surface	Enables precise cargo delivery and reduced off-target effects; customizable for various tumor targets	Fusion proteins may alter EV biogenesis; increased EV immunogenicity; efficacy dependent on recipient cells	[49,93,96,97,98,99,100,101]
**Modulation of EV Biogenesis**				
ESCRT-Dependent	Recruits ESCRT machinery to package cargo into vesicles	Exploits natural sorting pathways; high specificity for ubiquinated proteins	Possibility of cargo deubiquitinating prior to loading	[102,103,104,105,106,107,108]
ESCRT-Independent (Protein Loading)	Recruits tetraspanins to load/fuse cargo to the EV membrane	Enables engineered and inducible control of cargo; may be supported by cleavage sequences for intra-luminal loading	Fusion constructs may require significant optimization; proteins bound extra-luminally may be subject to degradation	[109,110,111,112,113,114,115,116,117]
ESCRT-Independent (RNA Loading)	Employing RNA-binding proteins and RNA sequence motifs for selective RNA packaging	Utilizes natural sorting mechanisms; efficient loading for large RNA-based cargo	Limited to RNA cargo compatible with recognition sequences; dependent on RNA cargo-RNA-binding protein affinity	[41,118,119,120,121,122,123,124,125,126,127,128]

Abbreviations: ESCRT: endosomal sorting complex required for transport; EVs: extracellular vesicles.

**Table 3:** Summary of strategies for EV surface engineering and cargo loading to boost anti-tumor immunity in breast cancers and beyond.

Cargo	Parental Cell	Recipient Cell	Reported Outcomes	Reference
cGAMP (STING agonist)	HEK293T	B16F10	Increased activation of CD8^+^ T cells, reduced tumor growth	[150]
Anti-IL-3R	Tumor-derived endothelial cells	MDA-MB-231	Reduced tumor cell viability, migration, and stemness markers, abolished metastasis from primary tumors	[151]
PD-L1 and Imiquimod	HEK293T	Mouse 4T1	Reversed CD8^+^ T cell exhaustion, enhanced tumor killing	[152]
Anti-PD-1	*Escherichia coli*	B16F10	Increased activation of tumor-specific CD8^+^ T cells	[153,154]
CD62L and CD40L	DC2.4 dendritic cells	Mouse 4T1	Increased expansion of CD8^+^ T cells, reduced activation of regulatory T cells	[155]
Anti-CD3 and anti-EGFR	Expi-293F	MDA-MB-468	Increased activation of CD8^+^ T cells, enhanced tumor killing	[156]
Anti-CD3 and anti-HER2	Expi-293F	SK-BR-3, HCC1954	Increased activation of CD8^+^ T cells, enhanced tumor killing	[157]
Trastuzumab-CAR or Cetuximab-CAR	CAR-T cells	MDA-MB-231, SK-BR-3	Enhanced tumor killing, improved safety profile relative to standard CAR-T therapy	[158]
Mesothelin-CAR	CAR-T cells	BT-549, mesothelin^+^ MDA-MB-231	Enhanced tumor killing	[159]
BCL-2 siRNA	NK92-MI	MCF-7	Induced apoptosis in tumor cells	[160]
Chlorin e6	NK92-MI	HepG2, CT26	ROS amplification induced M1-like macrophage polarization in RAW264.7 cells	[161]
HER2 and T7 Peptide-CAR	NK92-MI	JIMT-1	ROS amplification, successful blood–brain barrier transcytosis, enhanced HER2^+^ tumor killing	[162]
NKG2D and IL-24	CAR-NK92	MCF-7, HeLa, A549	Induced apoptosis in tumor cells	[163]
CRV peptide fused to Lamp2b	HEK293T	M2-like TAMs, LLC1	Induced M1-like macrophage polarization, increased T cell infiltration, decreased myeloid-derived suppressor cell activation	[164]
Anti-CD47 and anti-SIRPɑ	RAW264.7 macrophages	Mouse 4T1	Induced M1-like macrophage polarization, enhanced tumor killing	[165]
NF-κB p50 siRNA and miR-511-3p		Mouse 4T1	Induced M1-like macrophage polarization, enhanced tumor killing	[166]

Abbreviations: BCL-2: B cell lymphoma 2; CAR: chimeric antigen receptor; cGAMP: cyclic GMP-AMP; EGFR: epidermal growth factor receptor; HER2: human epidermal growth factor receptor 2; Lamp2b: lysosomal associated membrane protein 2b; NF-κB: nuclear factor kappa B; PD-L1: programmed death ligand 1; ROS: reactive oxygen species; siRNA: small interfering RNA; SIRPɑ: signal regulatory protein alpha; STING: stimulator of interferon genes; TAMs: tumor-associated macrophages.

## Abbreviations

The following abbreviations are used in this manuscript:

ALIX	Apoptosis Linked Gene 2-interacting protein X
cGAMP	Cyclic guanosine monophosphate-adenosine monophosphate
CSCs	Cancer stem cells
DARPin	Designed ankyrin repeat protein
ESCRT	Endosomal sorting complexes required for transport
EVs	Extracellular vesicles
hnRNPA1	Heterogenous nuclear ribonucleoprotein A1
ILV	Intraluminal vesicle
LNPs	Lipid nanoparticles
mRNA	Messenger ribonucleic acid
miRNA	Micro-ribonucleic acid
MSC	Mesenchymal stem cells
MVB	Multivesicular body
Ndfip1	Nedd4 Family-interacting protein 1
PTGFRN	Prostaglandin F2 Receptor Negative Regulator
siRNA	Small interfering ribonucleic acid
TAM	Tumor-associated macrophage
TNBC	Triple-negative breast cancer
TRAIL	Tumor necrosis factor-related apoptosis-inducing ligand
TSG101	Tumor susceptibility gene 101
UV	Ultraviolet
VEGFR1	Vascular endothelial growth factor receptor 1
VPS4	Vacuolar protein sorting 4
